# Tooth-Level Analysis of Dental Caries in Primary Dentition in Myanmar Children

**DOI:** 10.3390/ijerph17207613

**Published:** 2020-10-19

**Authors:** Yoshiaki Nomura, Ryoko Otsuka, Wit Yee Wint, Ayako Okada, Ryo Hasegawa, Nobuhiro Hanada

**Affiliations:** 1 Department of Translational Research, Tsurumi University School of Dental Medicine, Kanagawa 230-8501, Japan; otsuka-ryoko@tsurumi-u.ac.jp (R.O.); wityeewint14@gmail.com (W.Y.W.); hasegawakejp@gmail.com (R.H.); hanada-n@tsurumi-u.ac.jp (N.H.); 2 Department of Operative Dentistry, Tsurumi University School of Dental Medicine, Yokohama 230-8501, Japan; okada-a@tsurumi-u.ac.jp

**Keywords:** dental caries, primary dentition, school children, prevalence pattern, statistical modeling

## Abstract

In developing countries, the prevalence of dental caries in children remains high, which means that implementing a simple and convenient classification is critical. The classification needs to be evidence-based and needs to reflect tooth-level information. In this study, the prevalence of dental caries in the primary dentition of 352 Myanmar school children at the ages of 5, 6, and 7 was analyzed at the tooth level to clarify the underlying data structure of the patterns of dental caries in the population. Ninety-three percent of subjects had caries in primary dentition and the mean number of decayed teeth in primary dentition was 7.54 ± 4.82. Based on the item response theory analysis, mixed-effect modeling, and Bayesian network analysis, we proposed the following classification: Group 1: No dental caries; Group 2: Dental caries in molar teeth or dental caries in maxillary anterior teeth; Group 3: Dental caries in both molar and maxillary anterior teeth; Group 4: Dental carries in mandibular anterior teeth. Dental caries (dmft) in the groups was different between groups. The results of characteristics of tooth-level information and classification presented in this study may be a useful instrument for the analysis of the data of dental caries prevalence in primary dentition.

## 1. Introduction

Accumulated evidence and advanced prevention techniques of dental caries have contributed to the reduction in the prevalence of dental caries [1,2,3,4,5]; however, in developing countries, the prevalence of dental caries in children remains high and is one of the major public health concerns [6,7,8,9,10,11,12,13,14]. In these countries, dental treatment is still insufficient [15]. The prevalence pattern of dental caries varies with age and sex [16,17,18,19,20]. It depends on race, geographical location, socioeconomic status, nutrition habits, oral hygiene practices, and oral microbiome [21,22,23,24]. There are twenty teeth in the primary dentition. There is a clear prevalence pattern in the occurrence of dental caries in developing countries. To analyze the correlation of the prevalence pattern and its background factors, a simple and convenient index is indispensable.

Understanding the sophisticated patterns of the prevalence of dental caries leads to the classification of the subjects according to the severity of dental caries. Classification is useful for oral health instruction, treatment planning, and screening. It is also useful to summarize the severity of dental caries in epidemiological studies; however, most epidemiological studies present the prevalence of dental caries using summary statistics like the DMFT (Decayed Missing and Filled Teeth) index or descriptive statistics of the prevalence of dental caries. The DMFT index is a useful index that represents the prevalence of dental caries at the individual and community levels; however, the susceptibility of dental caries differs between the type of teeth and tooth surface. The DMFT index does not classify which tooth is affected by dental caries. In addition to the DMFT index, an index that reflects tooth-level information may be useful for the study of the etiology of dental caries, community-level surveys, and epidemiological studies. Tooth-level information enhances the investigator’s analysis ability.

Pioneering studies proposed primary dentition caries patterns [25,26,27,28,29,30,31]. These patterns were constructed by descriptive statistics or empirical evidence. Subsequently, several studies tried to identify the representative pattern by statistical modeling [32,33]; however, there has been no significant advance in this kind of classification study, even though various statistical modeling techniques are now available; therefore, a simple, easy, and user-friendly classification pattern for the surveillance and screening of dental caries is necessary. Data that show the prevalence of dental caries and the affected tooth are suitable. In advanced countries, the distribution of dental caries prevalence is skewed; modeling by the collection of rare cases is not appropriate.

In this study, the surveillance data of school children in Myanmar were analyzed at the tooth level to clarify the underlying data structure of patterns in the prevalence of dental caries in the primary dentition. In this study, we aim to find out the characteristics of dental caries prevalence in primary dentition at the tooth level and construct the practical classification pattern by statistical modeling.

## 2. Materials and Methods

### 2.1. Study Design

A cross-sectional survey was conducted to investigate the prevalence of dental caries in children in Myanmar.

### 2.2. Setting

Two schools located in the suburban area of the Naypyidaw district, the capital of Myanmar, were randomly selected from a list of schools. Oral examination was carried out from July to August 2019.

### 2.3. Participants

All of the Grade 0 and Grade 1 school children in two schools and Grade 2 children in one school were participants in the study. They were 5, 6, and 7 years old.

### 2.4. Oral Examination

Oral examinations were performed by two calibrated dentists working at the National Dental Hospital according to World Health Organization (WHO) standards. They were trained under WHO standard certified dentists at Niigata University, one of the WHO Collaboration Centers.

Dental caries conditions were recorded according to the WHO standard. Dental caries status of primary teeth was recorded as the following criteria: Sound, caries, restored, missing due to caries, or exfoliation. Permanent teeth were recorded as follows: Sound, dental caries, restored, or not erupted. Teeth with restorations were not observed. As this study was school-based, radiographs were not available.

### 2.5. Statistical Methods

#### 2.5.1. Item Response Theory

The three-parameter logistic model by item response theory (IRT) was used to identify items and calculate discriminations, difficulty, and guessing for each tooth type [34,35,36,37,38].

IRT analysis was carried out by R software Ver 3.50 with ltm, and the irtoys package by using the following formula:

(1) $$P_{i}\left(\theta \right)=\frac{\left(1-c_{i}\right)}{1+e^{-Da_{i}\left(\theta -b_{i}\right)}}$$

*a_i_*: Discrimination, *b_i_*: Difficulty, *c_i_*: Guessing

#### 2.5.2. Mixed-Effect Model

There are 20 teeth in one primary dentition. When tooth-level analysis is carried out, each tooth is not statistically independent, as each tooth is nested in a subject’s and tooth’s constructed hierarchy structure. The mixed-effect model was used to calculate the sensitivity for dental caries at the tooth level [39,40]. Sex and age were used for the subject-level parameter, and with or without dental caries by tooth was used for the tooth-levels parameter. A statistical model was constructed by the following model specification by IBM SPSS Statistics Ver 24.0 (IBM, Tokyo, Japan).

Data Structure: Subject, tooth

Probability distribution: Binominal

Link function: Normal

In what follows, fixed effects (resp. random effects or error terms) are denoted by Greek letters (resp. Alphabet)

Model

Subjects, tooth, and tooth surface are indexed by *_ijk_*.

(2) $$-\;L1:\;\;P_{o}{\left(\mathrm{dental}\;\mathrm{cries}\right)}_{ijk}=P_{o}(\mu_{ijk})=\pi_{0ijk}+\pi_{1jk}{\left(\mathrm{subject}\right)}_{ijk}+\varepsilon_{ijk}$$

(3) $$-\;L2:\;\;\pi_{0jk}=\;\;\beta_{00k}^{\left(m\right)}+\sum_{m=2}^{3}\beta_{001}^{\left(m\right)}{\left(\mathrm{dental}\;\mathrm{caries}\;\mathrm{indexed}\;\mathrm{by}\;m\right)}_{jk}+r_{0jk}$$

(4) $$-\;L2:\;\;\pi_{1jk}=\beta_{10k}$$

(5) $$\mathrm{where}\;\mathrm{dental}\;\mathrm{caries}\;\sim Bin\left(\alpha_{ijk},\tau_{ijk}\right)\;\mathrm{with}\;\mu_{ijk}=\alpha_{ijk}/\tau_{ijk}$$

(6) $$\;e_{ijk}\sim N\left(0,\delta_{e}^{2}\right),\;r_{0jk}\sim N\left(0,\delta_{r}^{2}\right),\mathrm{and}\;u_{01k}\sim N\left(0,\delta_{u^{\left(m\right)}}^{2}\right)$$

#### 2.5.3. Network Plot and Correspondence Analysis

The dataset of cross-tabulation within the prevalence of dental caries was used for network plot and correspondence analysis [40,41]. The network plot was performed using SPSS Modeler Ver.18.0 (IBM, Tokyo, Japan), and correspondence analysis was performed using SPSS Statistics Ver 24.0 (IBM, Tokyo, Japan).

#### 2.5.4. Bayesian Network

The dataset used for the Bayesian network analysis was the data dichotomous variable with or without dental caries in a group of teeth. The model was constructed by the tree-augmented naïve Bayes model. For the estimation, the maximum likelihood method was applied. The analysis was performed using SPSS Modeler Ver 18.0 (IBM, Tokyo, Japan) [42].

#### 2.5.5. Ordination Analysis

For the ordination analysis, a t-Distributed Stochastic Neighbor Embedding (tSNE) analysis was applied. tSNE analysis is a commonly used dimensionality reduction method, especially for bioinformatics [43]. The dataset used for the tSNE analysis was a dichotomous variable of each tooth with or without dental caries. tSNE analysis was performed using R software Ver 3.50 with vegan, and the Rtsne package.

### 2.6. Ethics

Informed consent was obtained by each child before the oral examination. This study was approved by the Ethical Committee of Tsurumi University School of Dental Medicine (Approval Number: 1624).

## 3. Results

### 3.1. Descriptive Statistics of the Subjects Participated in this Study and Their Teeth

In this study, we analyzed 199 male and 153 female elementary school children and graded either 0, 1, and 2. Grade and age correspond to Grade 0 for 5 years old, 1 for 6, and 2 for 7 in Myanmar. Ninety-three percent of subjects had caries in primary dentition and the dmft was 7.54 ± 4.82. A histogram of the number of dental caries is shown in Figure S1. A descriptive analysis of the dental caries is shown in Tables S1 and S2. The descriptive statistics of permanent teeth is shown in Table S3.

### 3.2. Tooth-Level Analysis of Dental Caries in Primary Dentition

#### 3.2.1. Analysis by Item Response Theory for the Susceptibility of Dental Caries in Primary Dentition

**Figure 1:**
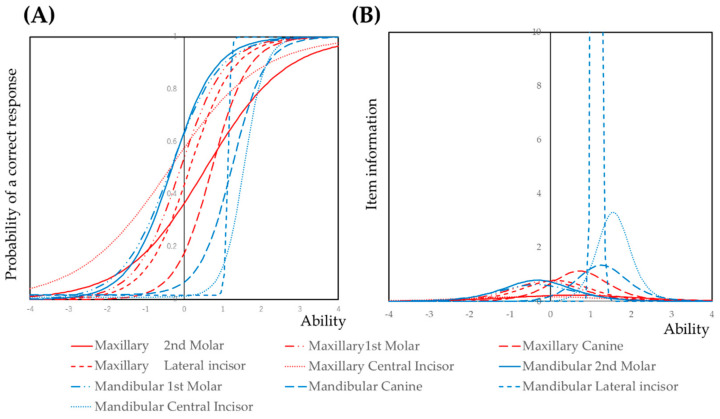
Item response curves and item information curves for the prevalence of dental caries. (**A**) Item response curve; (**B**) item information curve. In item response theory (IRT) analysis, the discriminatory power of an item is presented by the steepness of the curve at its inflection point and height of the item information curve. A steep curve and high item information indicate a distinction between respondents with low ability levels versus those with high ability levels. The item response curve and item information curve located in the forward direction indicated that subjects with high ability have a probability of a correct response. The item response curve and item information curve located in the backward direction indicate that even subjects with low ability can respond correctly. Susceptibility for dental caries was different between tooth types. The maxillary central incisor was found to often have dental caries. By contrast, the item response curve of the mandibular lateral incisor was steep and located in the forward direction, and the item information curve was high. It indicated that the mandibular lateral incisor often did not have dental caries. If dental caries was detected in the mandibular lateral incisor, most of the teeth were found to have dental caries in the primary dentition.

The prevalence of dental caries at the tooth level was analyzed using the three-parameter logistic model based on item response theory and mixed-effect modeling. The item response curve and item information curves are shown in Figure 1. The model is shown in Table S4.

For the mandibular teeth, item response curves of anterior teeth were shifted toward the forward direction; molar teeth were shifted toward the backward direction. The tendencies were not so clear when compared with maxillary teeth. The item information of the mandibular lateral incisor was extremely high and the peak was in the forward direction. It indicated that when dental caries is detected in the mandibular lateral incisor, the subject has many dental caries.

#### 3.2.2. Mixed-Effect Model Analysis of Susceptibility of Dental Caries in Primary Dentition

The result of the mixed-effect model analysis is shown in Table 1. When the mandibular central incisor was used as a reference, the maxillary central incisor was most susceptible to dental caries. Maxillary incisors and molars were susceptible for dental caries.

#### 3.2.3. Tooth-Level Co-Prevalence of Dental Caries in Primary Dentition

**Figure 2:**
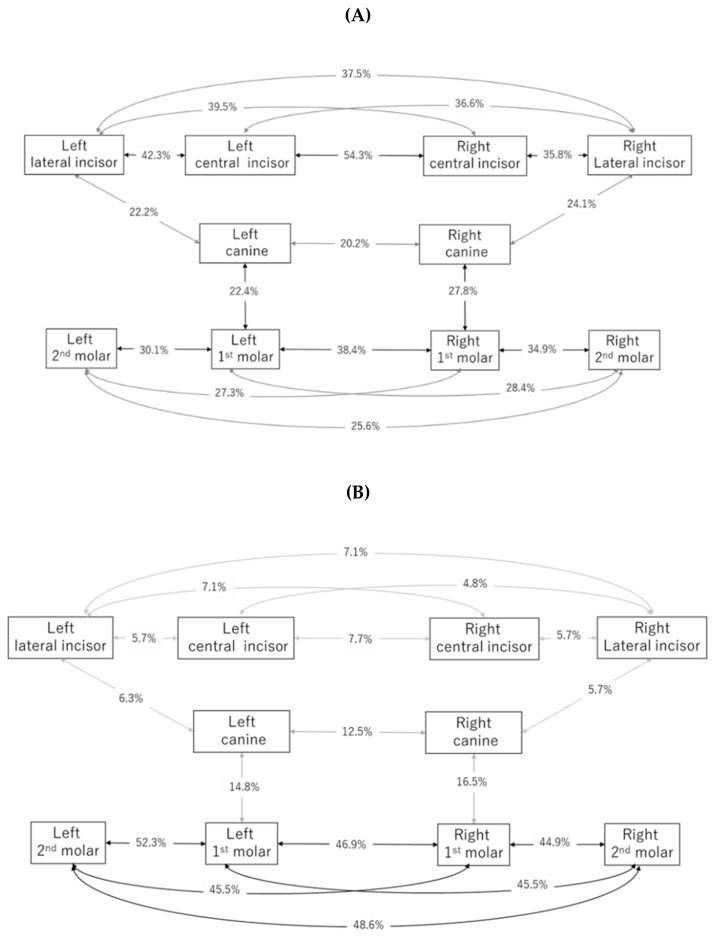
Network plot of co-incidence of dental caries. (**A**) Maxillary arch; (**B**) mandibular arch.

To find out the highly co-prevalent pattern of dental caries at the tooth level, a network plot was applied. The results are shown in Figure 2. Dental caries in maxillary central incisors were highly co-prevalent. More than 50% of subjects had dental caries in both of these teeth. More than 40% of the co-prevalence of dental caries occurred in mandibular molar teeth. The co-prevalence between adjacent teeth, right–left teeth, and counter-teeth is shown by the bar chart and shown in Figures S2–S4.

#### 3.2.4. Overview of Co-Prevalence of Dental Caries Teeth

**Figure 3:**
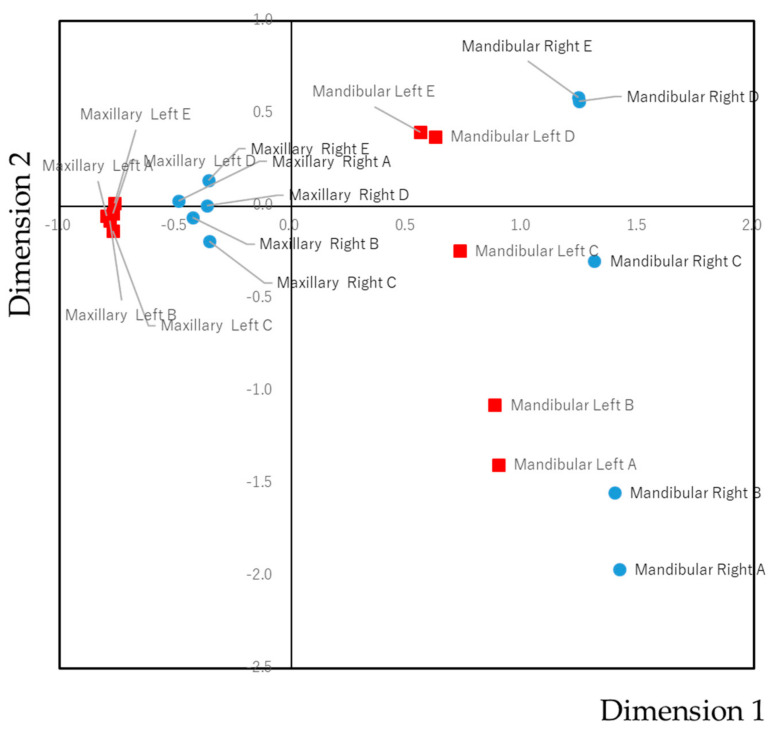
Biplot of the susceptibility of dental caries. The biplot graphically shows the correlations between multiple categorical variables. Items that had a strong correlation are closely located in the biplot. In this case, primary teeth with a high co-prevalence of dental caries in primary dentition are located closely.

To gain an overview of the tendencies of the co-prevalence of dental caries, including maxillary and mandibular arch correlation, a correspondence analysis was carried out (Figure 3). The spot of maxillary teeth was aggregated by the right and left side. For mandibular teeth, spots were located near the same side rather than the same kind of teeth. This indicated that dental caries tends to be co-prevalent on the unilateral rather than the bilateral or on the opposing teeth.

### 3.3. Classification of Prevalence of Dental Caries in Primary Dentition

#### 3.3.1. Regional Analysis for the Susceptibility of Dental Caries by Item Response Theory and Classification

Primary teeth were classified as maxillary or mandibular and anterior or molar. The prevalence of dental caries according to these blocks were analyzed by item response theory. The model is shown in Table S5.

**Figure 4:**
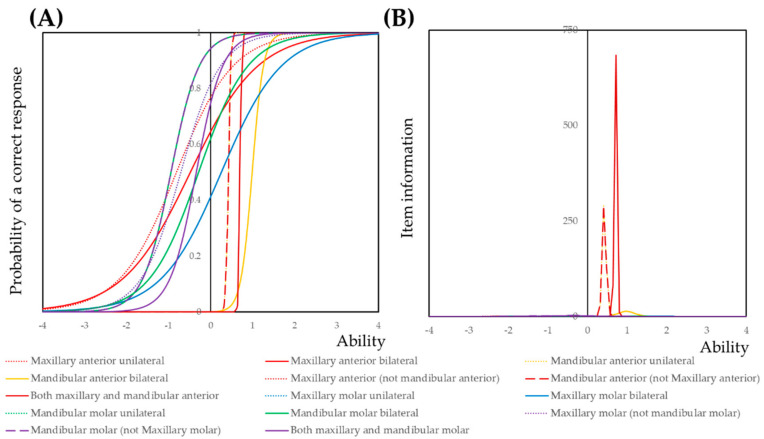
Item response theory analysis for the susceptibility of dental caries at the block level. (**A**) Item response curve; (**B**) item information curve; item information of dental caries in mandibular anterior teeth and both maxillary and mandibular anterior teeth had high item information.

The item information curve indicated that distinguishing between the unilateral or bilateral may not be a useful classification. Item information of dental caries in mandibular anterior teeth and both maxillary and mandibular anterior teeth had high item information (Figure 4). The peak of these item information curves shifted toward the forward side. By contrast, item information curves of molar teeth shifted toward the negative side. According to these results, we proposed four groups—Group 1: No dental caries, Group 2: Dental caries in molar teeth or dental caries in maxillary anterior teeth, Group 3: Dental caries in both molar and maxillary anterior teeth, Group 4: Dental caries in mandibular anterior teeth. Descriptive statistics against these groups were calculated. The result is shown in Table 2. *p*-value was statistically significant between all groups by multiple comparisons.

#### 3.3.2. Prevalence of Dental Caries by Conditional Probability

**Figure 5:**
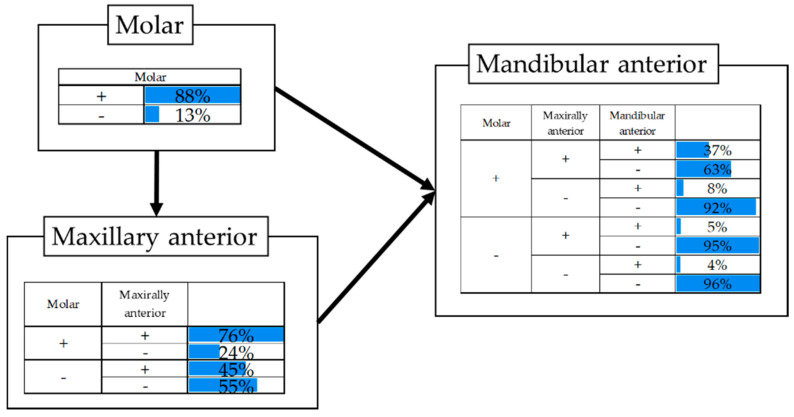
Bayesian network analysis of the conditional prevalence of dental caries.

The prevalence of dental caries in maxillary anterior teeth, mandibular anterior teeth, and molar teeth was analyzed by Bayesian network analysis (Figure 5). The result is shown in Figure 5. When dental caries existed in the maxillary anterior teeth, 92% of the subjects had dental caries in the molar teeth; however, for subjects who had dental caries both in the maxillary anterior teeth and molars, 37% of subjects had dental caries in mandibular anterior teeth. More than 90% of subjects who had neither dental caries in the maxillary anterior teeth nor molar had no dental caries in the mandibular anterior teeth.

#### 3.3.3. Ordination Analysis

**Figure 6:**
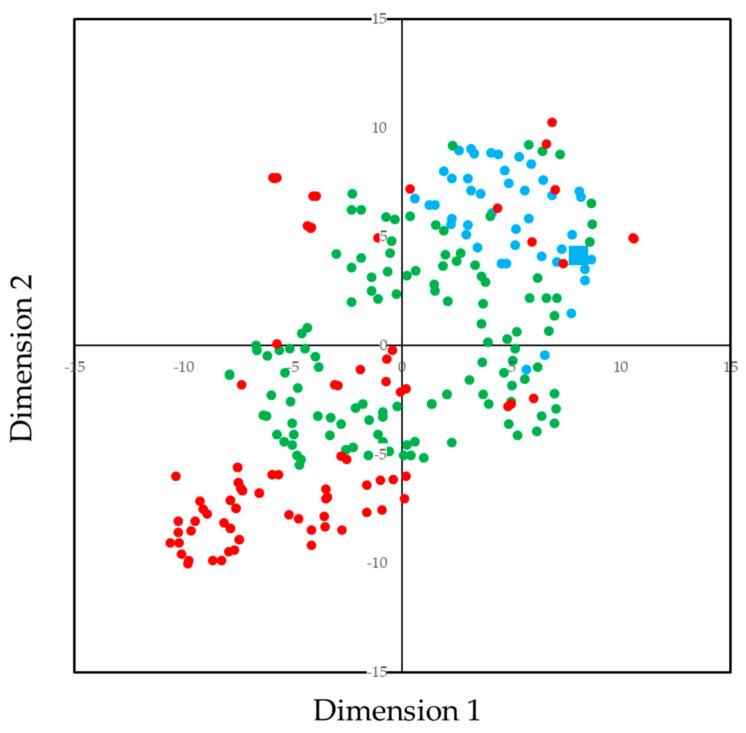
The t-Distributed Stochastic Neighbor Embedding (tSNE) plot for the prevalence of dental caries categorized by Groups. Group 1: No dental caries, Group 2: Dental caries in molar teeth or dental caries in maxillary anterior teeth, Group 3: Dental caries in both molar and maxillary anterior teeth, Group 4: Dental caries in mandibular anterior teeth. The tSNE analysis embeds high-dimensional data for visualization in a low-dimensional space of two dimensions. In this case, the prevalence of dental caries in 10 primary teeth was embedded in two dimensions independently of the Groups presented in Table 2. This reinforces the robustness of the groups presented in Table 2.

To confirm the validity of the classification presented in Table 2, a tSNE analysis was carried out by using the prevalence data of each teeth. The tSNE plot is shown in Figure 6. Some of the subjects who had dental caries in the mandibular anterior teeth were scattered, otherwise, spots by groups were aggregated.

## 4. Discussion

In this study, we investigated the prevalence of dental caries in primary dentition and implemented a classification of the pattern of dental caries in school children in Myanmar. Ninety-three percent of subjects had caries in primary dentition, and the mean number of decayed teeth in primary dentition was 7.54 ± 4.82. The prevalence of dental caries in primary dentition was extremely high when compared to another study [44]. A systematic review analyzed the worldwide prevalence of dental caries in primary dentition and found that the prevalence ranged from 23% to 90%. Among them, the prevalence of dental caries was higher in Asian countries. The highest prevalence was observed in the survey carried out in Indonesia [45]. The prevalence was 90% and the dmft was 7.5 ± 5.5—similar to our study [46]. Previous studies investigated preschool children at the age of 5. This age corresponded to Grade 0 in this study. The prevalence of this grade was 92.7% and dmft was 7.7 ± 5.1.

In this study, the prevalence of dental caries was analyzed at the tooth level and by tooth groups by several statistical modeling methods to confirm the robustness of the results. At the tooth level, coefficients by the mixed-effect model and the location of item response curves and item information curves were consistent. Item response cures of maxillary central incisors and mandibular molars are located in the backward direction (Figure 1). The coefficients of these teeth by mixed-effect modeling were higher than those of other teeth (Table 1). There are significant amounts of epidemiological data for the prevalence of dental caries. As far as we are aware, few reports analyzed tooth-level prevalence. Tooth-level data are nested in subjects; they are not statistically independent. Conventional statistical analysis is inclined to make type I errors [40]. Mixed-effect modeling and item response theory analysis are useful instruments for these nested data.

It has been reported that caries prevalence was higher in posterior teeth when compared with anterior teeth [47]; however, in this study, the highest prevalence of dental caries was found in maxillary central incisors. The lowest prevalence of dental caries was found in mandibular anterior teeth. The lowest prevalence in the maxillary arch was found in maxillary canine teeth. Prevalence differed by teeth. A rough classification of posterior teeth or anterior teeth may result in information loss. Previous reports had shown that higher caries prevalence was found in maxillary anterior teeth and mandibular posterior teeth [48]. These results are consistent with our study; however, in maxillary anterior teeth, only the central incisor had a higher prevalence when compared to maxillary molars. Prevalence in lateral incisors and canines was lower than that of the 1st molar. The difference in prevalence in maxillary anterior teeth was larger than those of other tooth groups.

In contrast to the discussion described above, simple, easy, and user-friendly classification is indispensable for the epidemiological studies. For the classification, minimum information loss and representing whole teeth are required.

Several reports attempted to classify the prevalence of dental caries. Symmetry analysis is a simple classification method. Tooth-level co-prevalence is basic data for symmetry analysis—shown in Figure 2. High co-prevalence occurred in mandibular molars followed by maxillary anterior teeth. The co-prevalence is affected by the prevalence of each tooth. Co-prevalence in symmetrical teeth is higher than adjacent teeth or counter-teeth. The co-prevalence by counter-teeth seems to be meaningless because of the difference in tooth-level prevalence, especially anterior teeth (Figure S4). The previous report had shown that dental caries in primary teeth shows high bilateral symmetry [49]. These reports had shown that the maxillary central incisor had higher bilateral co-prevalence. The results are consistent with our results and are expected due to the similar and higher prevalence in the maxillary central incisor; therefore, the highest co-prevalence was observed in the first molar in permanent teeth [49].

Patterns of dental caries in primary dentition have been presented [25,26,27,28,29,30,31]. The results by multidimensional analysis and cluster analysis also presented similar patterns [33,34]. These hierarchy patterns were similar to the pattern presented in this study; however, these patterns do not take into account dental caries in mandibular anterior teeth. The prevalence of dental caries in the area investigated in these studies was not high when compared with our study. For example, dmft in the highest dental caries prevalence group was 5.0. It may not be suitable to compare dental caries at the tooth and surface levels, and the value was lower than the dmft of the overall population of our study [28]. When applying these classifications, modification may be necessary to consider the additional patterns including dental caries in mandibular anterior teeth.

The limitation of this study is cross-sectional. More precise information may be obtained for the incidence of dental caries by a longitudinal study from infant age; however, no studies have confirmed the validity of a simple classification using tooth-level statistical modeling.

Clinicians or epidemiologists recognized the availability of simple classification following the severity of dental caries; however, the classification system was not generally applied for the evaluation of the severity of dental caries. For this purpose, the evidence is indispensable for the classification or indexes. The classification system presented in this study may be useful for epidemiological study or the screening of dental caries in primary dentition because of its simple and easy handling classification. The analysis presented in this study supports the evidence of this classification system in accordance with the severity of dental caries.

## 5. Conclusions

The results of the characteristics of tooth-level information and classification presented in this study can be a useful instrument for the surveillance to enhance the information.

## Figures and Tables

**Table 1:** Mixed-effect model analysis of the prevalence of dental caries at tooth level.

		Coefficient (95% CI)	*p*-Value
**Male/Female**		−0.028 (−0.052–−0.005)	0.019
Age		−0.013 (−0.027–0.001)	0.073
Maxillary	2nd Molar	0.274 (0.218–0.329)	<0.001
	1st Molar	0.429 (0.373–0.484)	<0.001
	Canine	0.184 (0.129–0.240)	<0.001
	Lateral incisor	0.390 (0.334–0.447)	<0.001
	Central Incisor	0.599 (0.541–0.657)	<0.001
Mandibular	2nd Molar	0.493 (0.438–0.549)	<0.001
	1st Molar	0.502 (0.446–0.557)	<0.001
	Canine	0.059 (0.004–0.115)	0.036
	Lateral incisor	−0.005 (−0.061–0.052)	0.872
	Central Incisor	Reference	
Intercept		0.210 (0.122–0.297)	<0.001

As teeth are nested in subjects, a mixed-effect model was applied for the analysis. Coefficients indicate the susceptibility of dental caries.

**Table 2:** The number of dental caries in the groups (dmft).

Group	*n*	Mean (SD)	Median (25th—75th)
1	23	0 ± 0	0 (0–0)
2	69	3.8 ± 2.1	4 (2–6)
3	165	7.3 ± 3.2	7 (5–10)
4	95	12.5 ± 4.1	12 (10–16)

The difference was statistically significant by the Kruskal–Wallis test. The difference between groups was all statistically significant by multiple comparisons of the Dann–Bonferroni method. Group 1: No dental caries, Group 2: Dental caries in molar teeth or dental caries in maxillary anterior teeth, Group 3: Dental caries in both molar and maxillary anterior teeth, Group 4: Dental caries in mandibular anterior teeth.

## Supplementary Materials

The following are available online at https://www.mdpi.com/1660-4601/17/20/7613/s1. Figure S1. Histogram of the number of dental caries. Figure S2. Co-prevalence of dental caries for adjacent teeth in the primary dentition. Figure S3. Co-prevalence of dental caries for symmetry teeth. Figure S4. Co-prevalence of dental caries for counter arch teeth. Table S1. The number of dental caries in primary dentition by grade and gender. Table S2. Tooth conditions in primary dentition analyzed in this study. Table S3. Descriptive statistics for permanent teeth. Table S4. IRT model for the prevalence of teeth in primary dentition. Table S5. IRT model for the prevalence pattern of dental caries in primary dentition.
