# Supplementary material for: Tooth-Level Analysis of Dental Caries in Primary Dentition in Myanmar Children
Source: Int J Environ Res Public Health. 2020 Oct 19;17(20):7613. doi: 10.3390/ijerph17207613 (PMC7589262; doi:10.3390/ijerph17207613)
Supplement: Supplementary file 1 [file ijerph-17-07613-s001.pdf]

International Journal of
Environmental Research and Public Health

Supplemental materials

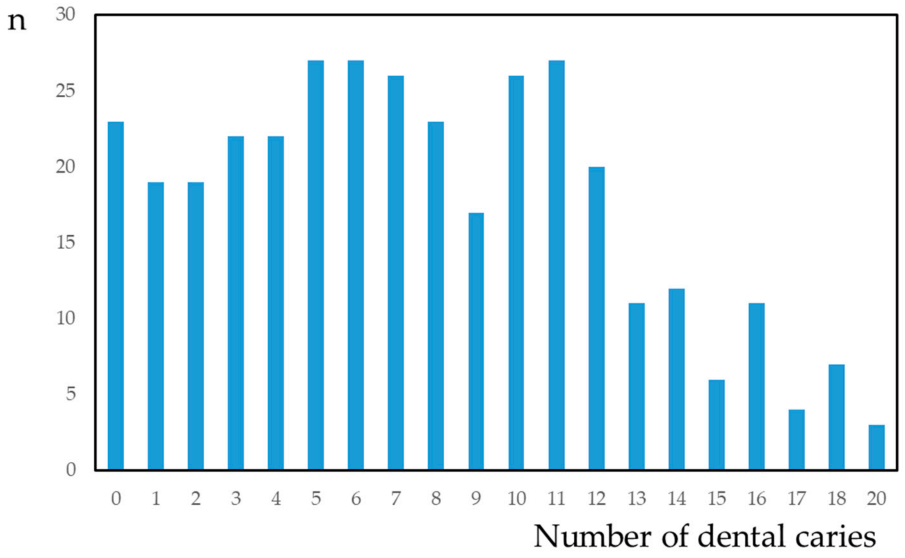

Figure S1. Histogram of number of dental caries.

Subjects with dental caries were 329 (93.5%). Prevalence of dental caries extremely high. As the missing teeth by dental caries was 58. They were included in this figure as dental caries. There was no filling teeth. By Kolmogorov–Smirnov test, number of dental carries was not normally distributed ($P < 0.001$).

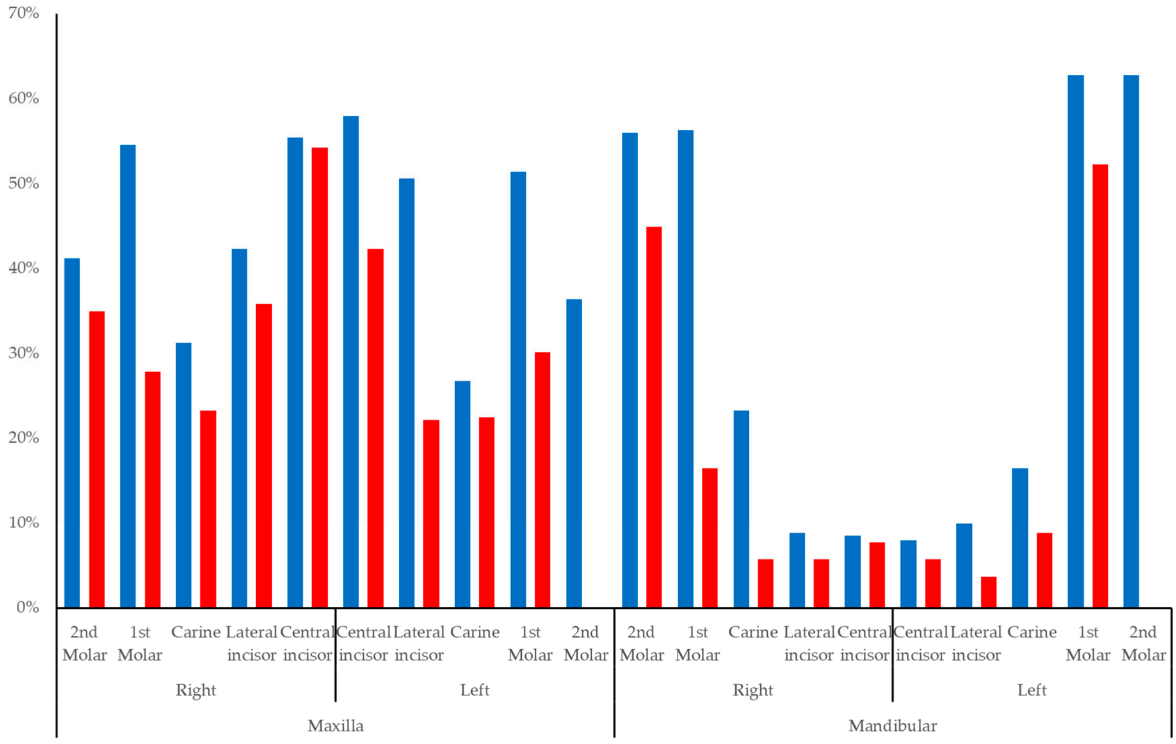

Figure S2. Co-prevalence of dental caries for adjacent teeth in primary dentition.

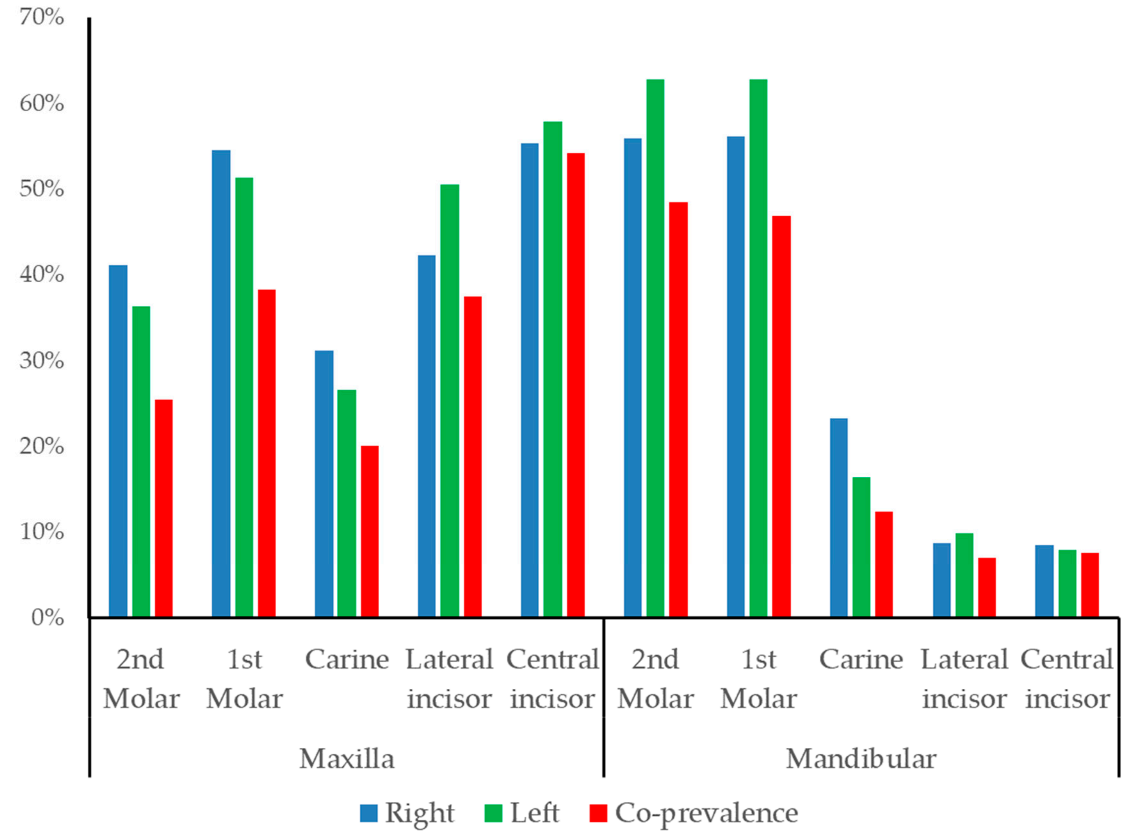

Figure S3. Co-prevalence of dental caries for symmetry teeth.

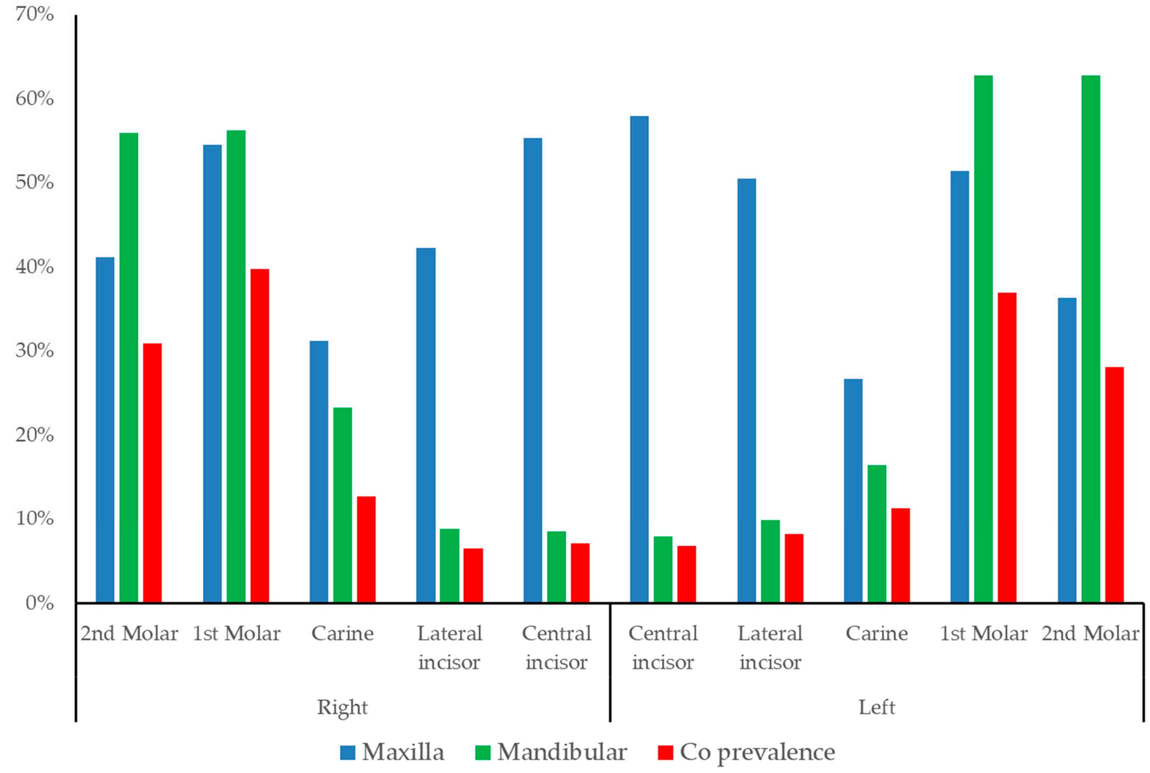

Figure S4. Co-prevalence of dental caries for counter arch teeth.

Table S1. Number of dental caries in primary dentition by grade and gender.

		Prevalence		dmft	
**Grade**	**Gender**	***n***	**%**	**Mean (SD)**	**Median (25th–75th)**
0	Male	94/95	98.9	8.1+/−5.0	8(4–11)
	Female	71/79	90.0	7.4+/−5.2	7(3–11)
	Total	165/178	92.7	7.7+/−5.1	7(4–11)
1	Male	78/82	95.1	7.5+/−4.6	7(4–11)
	Female	60/63	95.2	7.9+/−4.5	8(4–12)
	Total	138/145	95.2	7.7+/−4.8	7(4–11)
2	Male	17/18	94.4	5.6+/−3.4	6(3–8)
	Female	9/11	81.8	6.1+/−5.4	6(1–8)
	Total	26/29	89.7	5.8+/−4.2	6(2–8)

By the two way analysis of variance, age and gender were not statistically significant; *P*-values were for age, $P = 0.895$ for gender, $P = 0.239$ for grade, and $P = 0.551$ for interaction of age and gender.

Table S2. Tooth conditions in primary dentition analyzed in this study.

**Grade**		**Sound**	**Decayed**	**Missing**	**Deciduous**	**Total**
0	*n*	921	831	18	10	1780
	%	51.7%	46.7%	1.0%	0.6%	
1	*n*	710	625	14	101	1450
	%	49.0%	43.1%	1.0%	7.0%	
2	*n*	133	85	1	71	290
	%	45.9%	29.3%	0.3%	24.5%	
Total	*n*	1764	1541	33	182	3520
	%	50.1%	43.8%	0.9%	5.2%	

Table S3. Descriptive statistics for permanent teeth.

(A) Mean number of permanent teeth.

**Grade**	**Gender**	***n***	**Sound Teeth**		**Decayed Teeth (DMFT)**	
			**Mean (SD)**	**Median(25th–75th)**	**Mean (SD)**	**Median(25th–75th)**
0	Male	99	0.45+/−1.19	0(0–0)	0.06+/−0.6	0(0–0)
	Female	79	0.57+/−1.06	0(0–1)	0.01+/−0.11	0(0–0)
1	Male	82	3.79+/−3.29	3(1–6)	0.02+/−0.22	0(0–0)
	Female	63	3.56+/−2.48	4(1–5)	0.13+/−0.42	0(0–0)
2	Male	18	6.89+/−2.95	6.5(4–9)	0.06+/−0.24	0(0–0)
	Female	11	9.18+/−3.19	10(6–12)	0.27+/−0.65	0(0–0)

(B) Number of conditions of permanent teeth.

**Grade**	**Sound teeth**	**Decayed teeth**	**Not Erupted teeth**	**Total**
0	90	7	4709	4806
	1.9%	0.1%	98.0%	
1	536	9	3370	3915
	13.7%	0.2%	86.1%	
2	225	4	554	783
	28.7%	0.5%	70.8%	
Total	851	20	8633	9504
	9.0%	0.2%	90.8%	

Table S4. IRT model for the prevalence of teeth in primary dentition.

		Discrimination	Difficulty	Guessing
Maxilla	2nd Molar	0.97	0.56	<0.01
	1st Molar	1.74	−0.10	<0.01
	Canine	2.13	0.72	<0.01
	Lateral incisor	1.77	0.14	<0.01
	Central Incisor	0.85	−0.37	<0.01
Mandibular	2nd Molar	1.77	−0.32	<0.01
	1st Molar	1.58	−0.35	<0.01
	Canine	2.37	1.25	0.02
	Lateral incisor	26.22	1.13	0.02
	Central Incisor	3.68	1.55	0.01

Table S5. IRT model for the prevalence pattern of dental caries in primary dentition.

		Discrimination	Difficulty	Guessing
Anterior	Maxilla unilateral	1.48	−0.81	<0.001
	Maxilla bilateral	1.24	−0.48	<0.001
	Mandibular unilateral	36.87	0.42	<0.001
	Mandibular bilateral	7.42	0.99	<0.001
	Maxillary arch only	1.48	−0.81	<0.001
	Mandibular arch only	36.87	0.42	<0.001
	Both maxillary and mandibular arch	59.29	0.70	<0.001
Molar	Maxilla unilateral	2.11	−0.71	<0.001
	Maxilla bilateral	1.39	0.26	<0.001
	Mandibular unilateral	2.97	−0.95	<0.001
	Mandibular bilateral	1.70	−0.29	<0.001
	Maxillary arch only	2.11	−0.71	<0.001
	Mandibular arch only	2.97	−0.95	<0.001
	Both maxillary and mandibular arch	3.18	−0.34	<0.001
